# Serum uric acid to high-density lipoprotein cholesterol ratio is associated with stroke in the elderly: a population-based study

**DOI:** 10.3389/fneur.2025.1594080

**Published:** 2025-08-20

**Authors:** Qingsong Jiang, Guoyong Zhan, Yi Liu, Cai Jiang, Kang Wang, Guofu Zheng, Weixian Liu, Jiangchun Ma, Ming Wang, Zhuxiao Tang

**Affiliations:** ^1^Department of Neurosurgery, QuZhou KeCheng People’s Hospital, Quzhou, China; ^2^Department of Neurology, QuZhou KeCheng People’s Hospital, Quzhou, China; ^3^Brain Center, Zhejiang Hospital, Hangzhou, China

**Keywords:** serum uric acid, high-density lipoprotein cholesterol, ratio, stroke, NHANES

## Abstract

**Purpose:**

The ratio of serum uric acid (UA) to high-density lipoprotein cholesterol (HDL-c), known as UHR, has been identified as a novel marker for oxidative stress and metabolic disorders. This study focused on exploring the association between UHR and stroke risk among older adults in the United States.

**Methods:**

This cross-sectional study utilized data from individuals aged 60 years and older, collected through the 1999–2018 National Health and Nutrition Examination Survey (NHANES). Stroke assessment was based on participants’ self-reported history. The association between UHR and stroke risk was analyzed using logistic regression, restricted cubic splines (RCS), subgroup analysis, and receiver operating characteristic (ROC) curves.

**Results:**

This study included a total of 16,562 older adults, and the proportion of stroke cases increased with higher UHR levels. After adjusting for confounders, multivariable logistic regression analysis revealed that individuals in the highest UHR quartile had an odds ratio of 1.48 (1.23–1.80) for stroke risk compared to those in the lowest quartile. Subgroup analyses further demonstrated a stronger association in non-diabetic populations. RCS analysis suggested a linear relationship. Based on ROC results, UHR outperformed UA and HDL-c.

**Conclusion:**

Higher UHR levels are strongly associated with an increased risk of stroke in older adults. Additional large-scale prospective studies are required.

## 1 Introduction

With the global aging population on the rise, health issues among the elderly have become a critical focus in public health research (1). Stroke, as one of the leading causes of disability and mortality in older adults, continues to exhibit high incidence, death, and disability rates, imposing a substantial burden on healthcare systems worldwide (2, 3). Consequently, an in-depth investigation into the risk factors and underlying mechanisms of stroke is essential for developing effective prevention and treatment strategies.

The serum uric acid-to-high-density lipoprotein cholesterol ratio (UHR), an emerging composite indicator, might offer a more comprehensive reflection of the body’s metabolic state and oxidative stress levels (4, 5). Uric acid (UA), the final product of purine metabolism, is associated with various conditions, including hypertension, diabetes, and chronic kidney disease (6). Previous studies suggest that UA may directly contribute to atherosclerosis and thrombosis by promoting oxidative stress, inflammatory responses, and endothelial dysfunction, thereby elevating the risk of stroke (6–8). Conversely, high-density lipoprotein (HDL-c), often referred to as “good cholesterol,” is well-known for its cardiovascular protective effects, which are attributed to its roles in reverse cholesterol transport, as well as its anti-inflammatory and antioxidant properties (9, 10). The inverse relationship between these two parameters creates a synergistic effect where high UA combined with low HDL-c may amplify stroke risk beyond what either parameter would predict individually. Previous research has also highlighted that UHR, compared to individual markers, exhibits stronger associations with cardiometabolic diseases such as diabetes, hypertension, cardiovascular diseases, metabolic syndrome, and non-alcoholic fatty liver disease (NAFLD) (11–14).

Older adults frequently present a complex interplay of metabolic disorders, making them a distinct population for investigating multifactorial contributors to cerebrovascular risk. Notably, they are more susceptible to the concurrent presence of hyperuricemia and low HDL-c, both of which have independently been linked to elevated stroke risk. Despite the increasing incidence of stroke in this age group, most current risk-stratification tools are derived from general or mixed-age populations and may not adequately reflect the unique metabolic profiles of older adults. To date, no studies have specifically examined the association between the UHR and stroke in this demographic. Thus, this study seeks to address this gap by investigating the relationship between UHR and stroke among older adults in the United States, utilizing data from the National Health and Nutrition Examination Survey (NHANES). We hypothesize that elevated UHR levels are associated with increased stroke prevalence in older adults and may demonstrate stronger associations compared to UA or HDL-c alone.

## 2 Method

### 2.1 Data source

**Figure 1:**
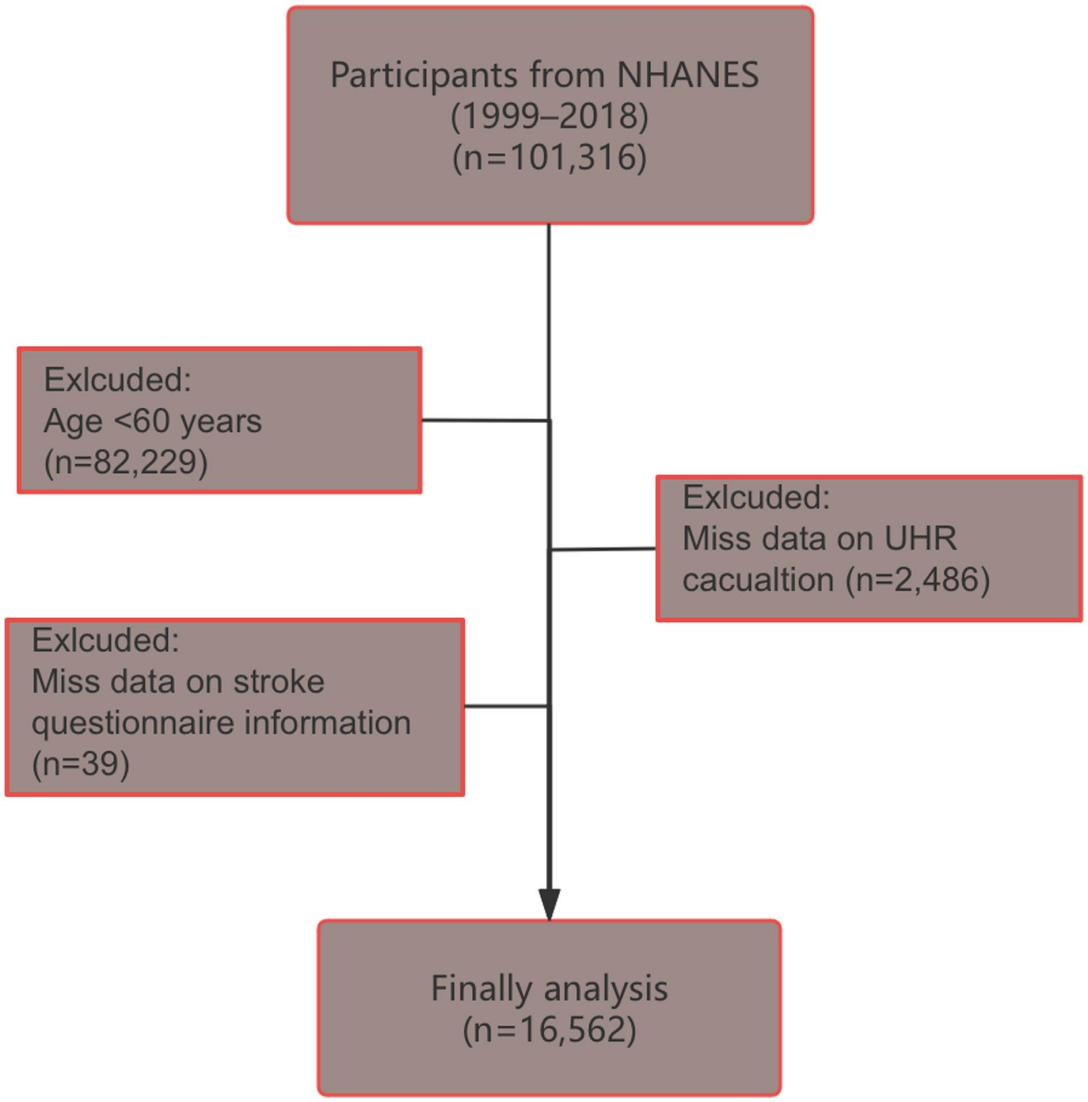
Flow chart of participant screening (selection process from NHANES 1999–2018 database to final study population of 16,562 older adults aged ≥60 years after excluding participants with missing UHR or stroke data).

NHANES, organized by the National Center for Health Statistics (NCHS) at the Centers for Disease Control and Prevention (CDC), used a multi-stage, stratified, and randomized sampling design to create a representative sample of the U. S. population.^1^ The study was approved by the NCHS Ethics Review Board,^2^ and written informed consent was obtained from all participants. This study analyzed data from 10 NHANES cycles (1999–2000 to 2017–2018). Of the original 101,316 participants, individuals younger than 60 years or missing UHR data or stroke questionnaire information were excluded, leaving a final analytical sample of 16,562 participants (Figure 1).

### 2.2 Definition of UHR and stroke

UHR, serving as the exposure in this study, was calculated by comparing UA (μmol/L) with HDL-c (mg/dL) (4, 15). UA levels were determined through enzymatic analysis using a DxC800 automated analyzer via the timed endpoint methodology. The process involved uricase-mediated oxidation of UA, generating allantoin and hydrogen peroxide as products. Subsequently, hydrogen peroxide underwent a peroxidase-catalyzed reaction with 4-aminoantipyrine (4-AAP) and 3,5-dichloro-2-hydroxybenzenesulfonate (DCHBS), yielding a chromogenic compound measured spectrophotometrically at 520 nm wavelength. For HDL-c quantification, magnesium sulfate/dextran solution was introduced to create water-soluble complexes with non-HDL-c fractions, preventing their interference in subsequent analytical steps. Polyethylene glycol esterase was then employed to hydrolyze HDL-c esters into free HDL-c. The resulting hydrogen peroxide reacted with 4-aminoantipyrine and HSDA, producing a purple-blue chromophore that was quantified photometrically at 600 nm. Participants were categorized into quartiles (Q1, Q2, Q3, and Q4) based on their UHR levels, with Q1 serving as the reference group. In our study, the definition of stroke was based on self-reported physician diagnosis from the NHANES database, and specific subtypes of stroke (ischemic vs. hemorrhagic) were not distinguished due to the database limitations (16–18).

### 2.3 Covariates

Our study controlled for various potential confounders, including demographic characteristics (age, sex, and ethnicity), socio-economic factors (education level and family income-to-poverty ratio (PIR)), health behaviors (smoking status), medical conditions (diabetes and hypertension), and medication use (antihypertensive, lipid-lowering, and urate-lowering drugs). Physiological and biochemical indicators such as body mass index (BMI), hemoglobin A1c (HbA1c), alanine aminotransferase (ALT), aspartate aminotransferase (AST), triglycerides (TG), total cholesterol (TC), low-density lipoprotein cholesterol (LDL-c), and serum creatinine (Scr) were also included. Ethnicity was classified into Mexican American, non-Hispanic White, non-Hispanic Black, other Hispanic, and other. Educational attainment was divided into three categories: less than high school, high school graduate, and college or higher. Smoking status was categorized as smokers (including both current and former smokers) versus non-smokers. Hypertension was defined as systolic blood pressure (SBP) ≥ 140 mmHg, diastolic blood pressure (DBP) ≥ 90 mmHg, self-reported diagnosis, or the use of antihypertensive medications. Diabetes was identified based on self-reported diabetes, use of insulin or hypoglycemic agents, HbA1c ≥ 6.5%, or fasting plasma glucose (FPG) ≥ 126 mg/dL. Medication use included antihypertensive, lipid-lowering, and urate-lowering drugs. History of medications was assessed by asking: “During the past 30 days, have you used or taken any prescription medications?”

### 2.4 Statistical analysis

Our analysis adhered to the NHANES guidelines, incorporating sample weights, clustering, and stratification to address the complex sampling design.^3^ Sample weighting was performed as recommended by NHANES, utilizing the formula Weights = 2/10 * WTMEC4YR + 1/10 * WTMEC2YR. We employed random forest-based iterative imputation to handle incomplete covariate data. Continuous variables were presented as means with standard deviations (SD), while categorical variables were described as counts with weighted percentages. Missing data were assumed to occur randomly, and the random forest algorithm was utilized for iterative imputation of missing covariates. Group comparisons were performed using the Kruskal-Wallis test and the Rao-Scott chi-square test. Logistic regression models were employed to examine the association between UHR (continuous/quartile) and stroke risk across three models (Model 1–3). Model 1 included no covariate adjustments, Model 2 adjusted for age, sex, and ethnicity, and Model 3 further adjusted for additional factors such as education level, PIR, smoking status, hypertension, diabetes, medication use (antihypertensive, lipid-lowering, and urate-lowering drugs), BMI, HbA1c, ALT, AST, TG, LDL-c, and Scr. Restricted cubic spline (RCS) analysis was applied to explore potential nonlinear relationships. Spearman correlation analysis was conducted to evaluate the correlation between UHR and other parameters. Stratified analyses were conducted to assess effect modifiers, including age, sex, ethnicity, NHANES cycles, BMI, hypertension, and diabetes. Receiver operating characteristic (ROC) curve analysis were used to evaluate the predictive performance of UHR for stroke. All statistical analyses were performed using R software (version 4.2.0), with statistical significance defined as *p* < 0.05.

## 3 Results

### 3.1 Baseline characteristics of study population

A total of 16,562 older adults participated in the study, with an average age of 70.66 years. Among them, 2,489 (weighted 4.07%) were Mexican American, 3,128 (weighted 8.30%) were non-Hispanic Black, 8,602 (weighted 78.76%) were non-Hispanic White, 1,259 (weighted 3.28%) were other Hispanic, and 1,084 (weighted 5.58%) were classified as other racial groups. Table 1 summarizes the baseline characteristics by UHR quartiles. Participants in the higher quartiles were predominantly male, had lower levels of PIR and education, smoked more frequently, and exhibited higher BMI, increased medication usage (including antihypertensive, lipid-lowering, and urate-lowering drugs), as well as higher proportions of hypertension and diabetes, compared to those in the lowest quartile (*p* < 0.001). Significant differences were observed in biochemical parameters such as HbA1c, ALT, TG, TC, HDL-c, LDL-c, SCr, and UA across quartiles (*p* < 0.001). Furthermore, the proportion of stroke cases increased with higher UHR levels (*p* < 0.001).

**Table 1:** Baseline characteristics based on UHR quartiles (Q1-Q4).

Variables	Overall	Quartile 1	Quartile 2	Quartile 3	Quartile 4	*p* value
Age (years)	70.66 ± 7.39	70.92 ± 7.51	70.69 ± 7.40	70.37 ± 7.29	70.66 ± 7.33	0.092
Sex						<0.001
Female	8,310 (54.83%)	3,125 (80.47%)	2,355 (60.65%)	1,708 (43.39%)	1,122 (29.23%)	
Male	8,252 (45.17%)	1,004 (19.53%)	1,786 (39.35%)	2,442 (56.61%)	3,020 (70.77%)	
Ethnicity						0.001
Mexican American	2,489 (4.07%)	582 (3.58%)	641 (4.27%)	674 (4.58%)	592 (3.93%)	
Non-Hispanic Black	3,128 (8.30%)	765 (7.92%)	823 (9.28%)	780 (8.18%)	760 (7.86%)	
Non-Hispanic White	8,602 (78.76%)	2,222 (80.97%)	2,046 (77.24%)	2,113 (77.90%)	2,221 (78.61%)	
Other Hispanic	1,259 (3.28%)	297 (2.94%)	358 (3.78%)	295 (3.15%)	309 (3.30%)	
Other Races	1,084 (5.58%)	263 (4.60%)	273 (5.42%)	288 (6.20%)	260 (6.30%)	
PIR	2.49 ± 1.48	2.61 ± 1.51	2.47 ± 1.48	2.50 ± 1.49	2.39 ± 1.43	<0.001
Education level						<0.001
Less than High school	5,688 (20.35%)	1,263 (16.56%)	1,450 (21.43%)	1,449 (21.06%)	1,526 (23.09%)	
High school	3,915 (25.81%)	943 (24.87%)	985 (26.59%)	992 (25.23%)	995 (26.74%)	
Some college or above	6,959 (53.84%)	1,923 (58.57%)	1,706 (51.99%)	1,709 (53.71%)	1,621 (50.17%)	
Smoking status						<0.001
No	8,000 (48.89%)	2,353 (55.97%)	2,077 (50.91%)	1,932 (47.24%)	1,638 (39.76%)	
Yes	8,562 (51.11%)	1,776 (44.03%)	2,064 (49.09%)	2,218 (52.76%)	2,504 (60.24%)	
BMI (kg/m*^2^*)	28.87 ± 5.98	26.33 ± 5.36	28.55 ± 5.82	29.76 ± 5.88	30.84 ± 5.89	<0.001
HbA1c (%)	6.05 ± 1.14	5.85 ± 1.11	6.01 ± 1.09	6.10 ± 1.14	6.24 ± 1.17	<0.001
ALT (U/L)	22.34 ± 20.96	20.50 ± 11.16	22.21 ± 33.61	22.53 ± 11.75	24.09 ± 18.94	<0.001
AST (U/L)	25.09 ± 17.29	24.80 ± 10.05	25.21 ± 28.24	24.71 ± 9.81	25.65 ± 14.14	0.053
TG (mg/dL)	154.89 ± 105.79	110.11 ± 57.66	136.77 ± 78.24	162.75 ± 100.85	209.79 ± 140.45	<0.001
TC (mg/dL)	196.65 ± 43.68	208.80 ± 40.15	199.08 ± 42.20	192.71 ± 43.28	186.04 ± 45.64	<0.001
HDL-c (mg/dL)	54.11 ± 16.60	72.33 ± 16.07	57.02 ± 10.59	48.21 ± 8.40	38.95 ± 7.53	<0.001
LDL-c (mg/dL)	113.98 ± 35.56	116.44 ± 33.18	116.47 ± 35.69	114.01 ± 35.92	109.01 ± 36.84	<0.001
SCr (μmol/L)	89.19 ± 51.19	75.20 ± 42.71	84.51 ± 47.12	91.37 ± 48.21	105.62 ± 60.18	<0.001
UA (μmol/L)	5.72 ± 1.50	4.36 ± 0.96	5.32 ± 0.96	6.01 ± 1.02	7.19 ± 1.35	<0.001
UHR	6.99 ± 3.12	3.67 ± 0.74	5.57 ± 0.50	7.45 ± 0.63	11.24 ± 2.57	<0.001
Hypertension, %						<0.001
No	5,202 (33.49%)	1,551 (41.18%)	1,350 (35.76%)	1,241 (31.10%)	1,060 (24.13%)	
Yes	11,360 (66.51%)	2,578 (58.82%)	2,791 (64.24%)	2,909 (68.90%)	3,082 (75.87%)	
Diabetes, %						<0.001
No	11,560 (74.13%)	3,355 (86.44%)	2,966 (76.57%)	2,791 (70.85%)	2,448 (59.87%)	
Yes	5,002 (25.87%)	774 (13.56%)	1,175 (23.43%)	1,359 (29.15%)	1,694 (40.13%)	
Medication use, %						
Antihypertensive	9,363 (57.43%)	1,904 (44.97%)	2,242 (54.94%)	2,420 (59.94%)	2,797 (72.74%)	<0.001
Lipid-lowering	6,292 (42.88%)	1,269 (33.46%)	1,562 (42.11%)	1,694 (47.34%)	1,767 (50.54%)	<0.001
Urate-lowering	507 (3.21%)	80 (2.02%)	105 (2.41%)	145 (3.30%)	177 (5.44%)	<0.001
Stroke, %						<0.001
No	15,175 (92.39%)	3,869 (94.44%)	3,818 (92.60%)	3,797 (92.45%)	3,691 (89.56%)	
Yes	1,387 (7.61%)	260 (5.56%)	323 (7.40%)	353 (7.55%)	451 (10.44%)	

PIR, family income-to-poverty ratio; BMI, body mass index; HbA1c, hemoglobin A1c; ALT, alanine aminotransferase; AST, aspartate aminotransferase; TG, triglycerides TC, total cholesterol; HDL-c, high-density lipoprotein cholesterol; LDL-c, low-density lipoprotein cholesterol; Scr, serum creatinine; UA, uric acid; UHR, serum uric acid to high-density lipoprotein cholesterol.

### 3.2 Association between UHR and stroke

**Figure 2:**
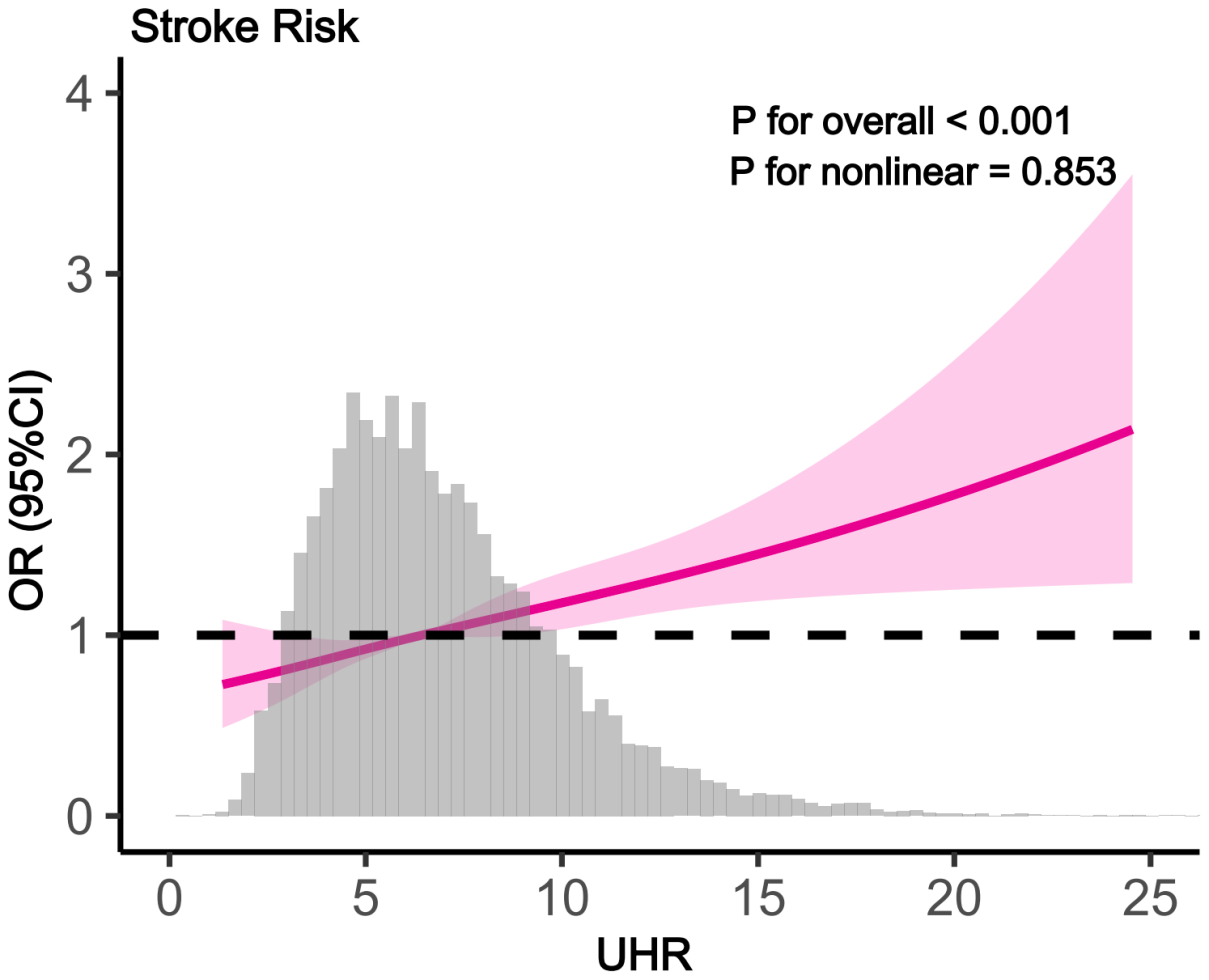
The results of RCS analysis (RCS showing linear positive association between UHR and stroke odds ratio (95% CI). Gray histogram shows UHR distribution. P for overall <0.001, P for nonlinear = 0.853).

To explore the independent relationship between UHR and stroke risk, we constructed three logistic regression models (Table 2). In the unadjusted analysis, the odds ratios (ORs) and 95% confidence intervals (CIs) for stroke across quartiles were 1.00 (reference), 1.26 (1.06–1.49), 1.38 (1.17–1.63), and 1.82 (1.55–2.13). After adjusting for age, sex, and ethnicity in Model 2, a significant trend of increasing stroke risk with higher UHR levels was observed (*P* for trend<0.001). In Model 3, which included additional adjustments for education level, PIR, smoking status, hypertension, diabetes, medication use, BMI, HbA1c, ALT, AST, TG, LDL-c, and Scr, the ORs and 95% CIs for stroke risk were 1.00 (reference), 1.14 (0.95–1.36), 1.21 (1.01–1.45), and 1.42 (1.17–1.73) across quartiles, with a trend test *p* < 0.001. When UHR was analyzed as a continuous variable, each 1-unit increase in UHR was associated with 5% higher odds of stroke after full adjustment (Model 3: OR = 1.05, 95% CI: 1.03–1.07, *p* < 0.001). RCS analysis further supported a linear positive relationship between UHR and stroke risk, with no evidence of a nonlinear pattern (***P*** for nonlinear = 0.598) (Figure 2).

**Table 2:** Logistic regression analysis results of UHR and stroke risk.

Variables	OR (95%CI) *p* value		
	Model 1	Model 2	Model 3
Continuous	1.07 (1.06, 1.09) < 0.001	1.08 (1.06, 1.09) < 0.001	1.05 (1.03, 1.07) < 0.001
Categories			
Quantile 1	reference	reference	reference
Quantile 2	1.26 (1.06, 1.49) 0.008	1.28 (1.08, 1.52) 0.004	1.14 (0.95, 1.36) 0.151
Quantile 3	1.38 (1.17, 1.63) < 0.001	1.44 (1.22, 1.71) < 0.001	1.21 (1.01, 1.45) 0.039
Quantile 4	1.82 (1.55, 2.13) < 0.001	1.88 (1.59, 2.23) < 0.001	1.42 (1.17, 1.73) < 0.001
*P* for trend	<0.001	<0.001	<0.001

OR, odds ratio; 95% CI, 95% confidence interval.

Model 1: no adjusted.

Model 2: adjusted for age, sex, and ethnicity.

Model 3: adjusted for age, sex, ethnicity, education level, PIR, smoking status, hypertension, diabetes, medication use (antihypertensive, lipid-lowering, and urate-lowering drugs), BMI, HbA1c, ALT, AST, TG, LDL-c, and Scr.

### 3.3 Correlation of UHR with clinical parameters

Spearman correlation analysis revealed that UHR exhibited consistent positive correlations with smoking status, BMI, HbA1c, ALT, TG, SCr, UA, hypertension, diabetes, medication use, and stroke, and negative correlations with PIR, education level, TC, HDL-c, and LDL-c (*p* < 0.01) (Table 3). These associations remained significant both before and after adjustments for age, sex, and ethnicity (*p* < 0.01).

**Table 3:** The results of correlation analysis.

Variables	Non-adjusted	Adjusted for age, sex, and ethnicity
Age (years)	−0.013	–
Sex	0.375**	–
Ethnicity	−0.002	–
PIR	−0.049**	−0.085**
Education level	−0.054**	−0.059**
Smoking status	0.129**	0.032**
BMI (kg/m*^2^*)	0.317**	0.266**
HbA1c (%)	0.199**	0.121**
ALT (U/L)	0.126**	0.028**
AST (U/L)	0.006	0.007
TG (mg/dL)	0.420**	0.390**
TC (mg/dL)	−0.217**	−0.139**
HDL-c (mg/dL)	−0.809**	−0.700**
LDL-c (mg/dL)	−0.094**	−0.055**
SCr (μmol/L)	0.413**	0.182**
UA (μmol/L)	0.739**	0.718**
Hypertension	0.099**	0.139**
Diabetes	0.180**	0.171**
Antihypertensive drugs	0.160**	0.185**
Lipid-lowering drugs	0.092**	0.067**
Urate-lowering drugs	0.054**	0.025**
Stroke	0.064**	0.068**

**p < 0.01.

### 3.4 Subgroup analysis and ROC analysis

**Figure 3:**
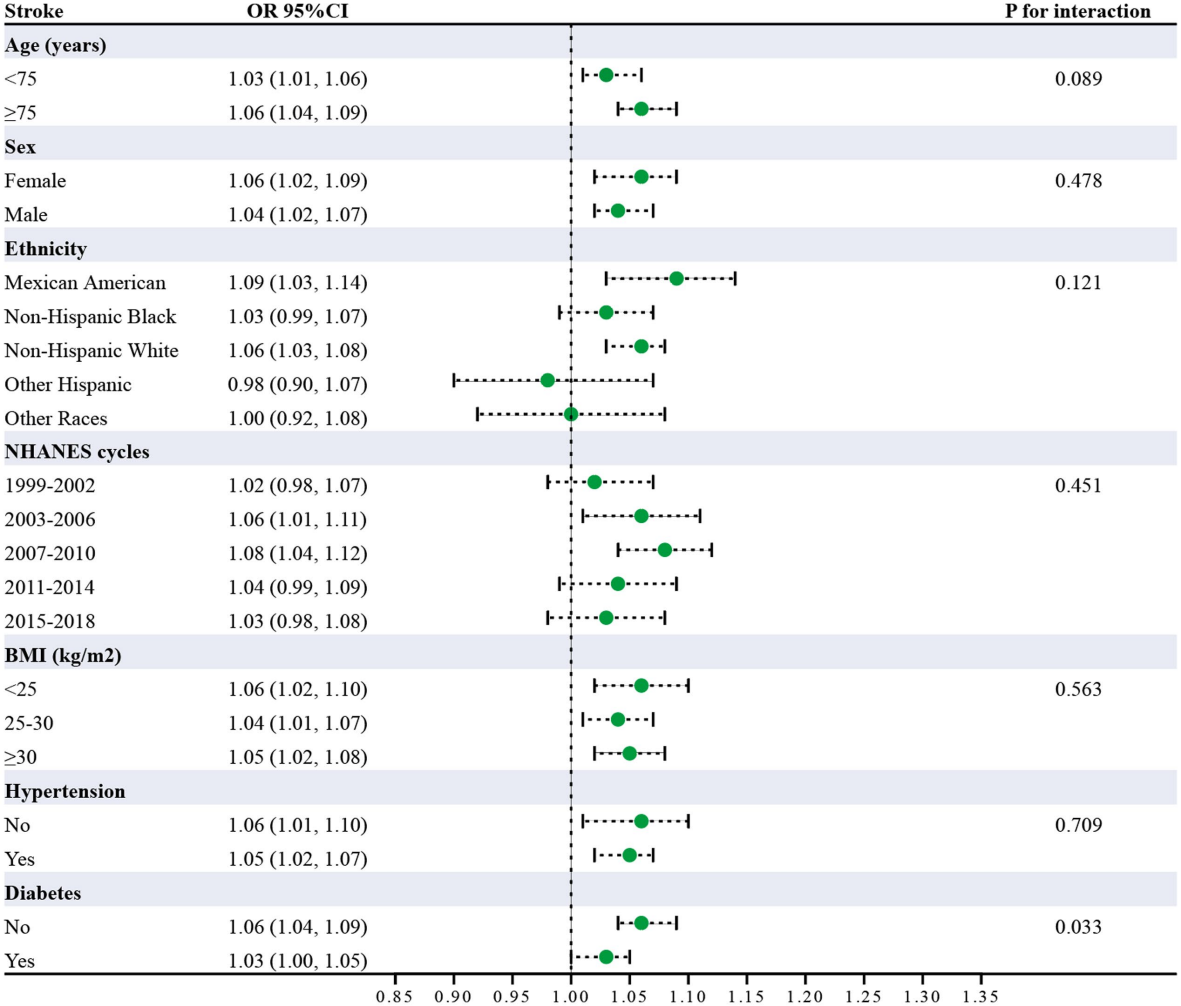
Subgroup analysis of UHR and stroke risk (Odds ratios (95% CI) for stroke per unit UHR increase across demographic and clinical characteristics. Diabetes showed significant interaction (*p* = 0.033). All analyses adjusted for confounders).

**Figure 4:**
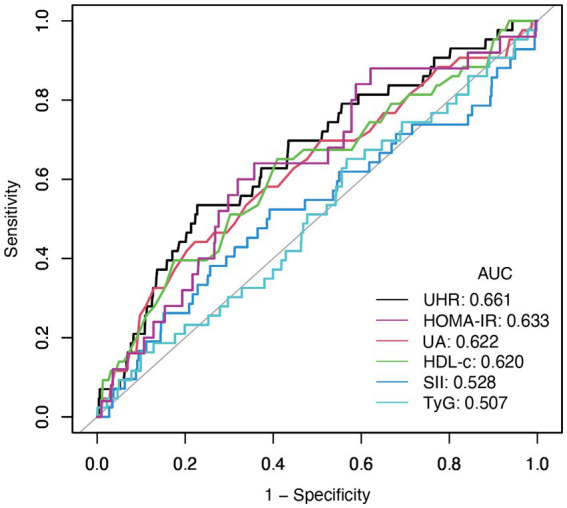
The results of ROC analysis (UHR showed highest AUC (0.661) compared to UA (0.622), HDL-c (0.620), HOMA-IR (0.633), SII (0.528), and TyG (0.507) for stroke risk discrimination).

Subgroup analysis was carried out to investigate the link between UHR and stroke risk, stratified by variables such as age, sex, ethnicity, NHANES cycles, BMI, hypertension, and diabetes (Figure 3). The analysis showed that diabetes status significantly impacted these associations (*P* for interaction <0.05). Interaction analysis highlighted that the association between UHR and stroke was stronger in non-diabetic individuals (OR = 1.06; 95%CI = 1.04–1.09) than in diabetic individuals (OR = 1.03; 95%CI = 1.00–1.05). We compared the discriminative value of UHR with different markers of inflammation [systemic immune-inflammation index (SII)] (19), metabolic disorders [triglyceride-glucose index (TyG)] (17), and insulin resistance [homeostatic model assessment of insulin resistance (HOMA-IR)] (20) for stroke. According to ROC analysis, UHR demonstrated superior predictive performance compared to other indices, with AUC values of 66.1% for UHR, 62.2% for UA, 62.0% for HDL-c, 52.8% for SII, 50.7% for TyG, and 63.3% for HOMA-IR (Delong test: *p* < 0.001) (Figure 4; Table 4).

**Table 4:** The sensitivity and specificity values of ROC results.

Stroke	AUC	95%CI lower	95%CI upper	Best threshold	Specificity	Sensitivity
UHR	0.661	0.576	0.747	8.725	0.773	0.535
UA	0.622	0.529	0.714	389.600	0.778	0.442
HDL-c	0.620	0.528	0.713	47.500	0.591	0.651
SII	0.528	0.427	0.629	385.479	0.608	0.524
TyG	0.507	0.417	0.598	8.801	0.433	0.651
HOMA-IR	0.633	0.520	0.747	3.546	0.643	0.640

## 4 Discussion

This study, utilizing cross-sectional data from an elderly population, is the first to investigate the association between the UHR and stroke risk in older adults. The findings reveal that an elevated UHR is significantly associated with an increased risk of stroke in this population. Subgroup analyses further indicate that this relationship is more pronounced in non-diabetic individuals. ROC analysis demonstrates that the diagnostic value of UHR surpasses that of UA or HDL-c alone.

Previous studies have predominantly focused on single indicators, often neglecting the interaction between UA and HDL-c and the potential clinical significance of their ratio. For example, hyperuricemia has been consistently linked to an elevated risk of stroke, particularly in elderly populations (21, 22). Similarly, low HDL-c levels have been identified as an independent risk factor for stroke (23). However, evidence suggests an antagonistic relationship between UA and HDL-c in the context of metabolism and cardiovascular diseases. First, as the product of purine metabolism, elevated UA levels can induce oxidative stress and endothelial dysfunction, thereby promoting atherosclerosis. Research has shown that high UA levels are associated with reduced vascular endothelial nitric oxide (NO) production, which may lead to vasoconstriction, increased blood pressure, and thrombosis, ultimately heightening stroke risk (8, 24, 25). Second, HDL-c exerts protective effects through its anti-inflammatory, antioxidant, and reverse cholesterol transport functions. By removing cholesterol from vascular walls and inhibiting the oxidation of LDL-c, HDL-c helps preserve endothelial function (26). A reduction in HDL-c levels diminishes this protective effect (26). When UA levels are elevated and HDL-c levels are decreased, their combined effects may exacerbate vascular damage and further increase stroke risk. Additionally, an elevated UHR might also reflect the presence of metabolic syndrome, which is characterized by insulin resistance, hypertension, obesity, and dyslipidemia-all of which are closely associated with stroke risk (27). On the other hand, the stronger association in non-diabetic populations may be attributed to several potential mechanisms that require further investigation. First, diabetic patients already have elevated baseline stroke risk due to existing vascular complications, which may obscure the independent predictive value of UHR. Second, diabetic patients may have developed severe metabolic dysfunction that could mask the additional risk contribution of UHR. However, the exact mechanisms underlying this difference remain unclear and warrant validation. Compared to analyzing UA or HDL-c individually, UHR may offer greater clinical utility. On one hand, UHR reflects the combined effects of oxidative stress and metabolic dysfunction. On the other hand, its calculation is straightforward and easily applicable in clinical practice. Therefore, this study provides novel evidence supporting UHR as a predictive marker for stroke risk (28).

This study identified a significant association between the UHR and stroke risk in older adults; however, several limitations should be acknowledged. First, as a cross-sectional study, it cannot establish causality or determine the temporal sequence between UHR elevation and stroke occurrence. The elevated UHR observed in stroke patients could potentially be a consequence of the stroke itself rather than a causal factor. The study by Ding et al. (29), demonstrating UHR’s association with post-stroke mortality, provides complementary evidence. These findings may suggest UHR reflects underlying metabolic dysfunction relevant to post-stroke outcomes (29). Additionally, previous studies have shown UHR associations with cardiovascular risk factors linked to stroke. While mediation analysis could explore these pathways, our cross-sectional design and lack of temporal data precluded such analysis. Second, the data were obtained from a specific population, which may introduce selection bias, limiting the generalizability of the results to other populations. Third, the study did not fully account for other potential confounding factors, such as dietary habits and the use of lipid-lowering medications, which could have influenced the observed outcomes. Finally, it should be noted that although our study and some previous reports suggest a correlation between UHR and various cardiometabolic diseases, there is still a lack of large-scale prospective clinical studies to confirm the diagnostic or predictive value of UHR. Currently, UHR is not recommended by clinical guidelines as a routine marker, and its clinical utility requires further validation through multi-center and large cohort studies.

## 5 Conclusion

Our study found that higher UHR levels are significantly associated with increased stroke risk in older adults, particularly in non-diabetic individuals. UHR showed better predictive value for stroke risk compared to UA or HDL-c alone. This research underscores the importance of composite biomarkers in assessing stroke risk.

## Data Availability

Publicly available datasets were analyzed in this study. This data can be found at: https://wwwn.cdc.gov/nchs/nhanes.

## Footnotes

^1^ https://wwwn.cdc.gov/nchs/nhanes/

^2^ https://www.cdc.gov/nchs/nhanes/about/erb.html

^3^ https://wwwn.cdc.gov/nchs/nhanes/analyticguidelines.aspx
